# Establishment, Prediction, and Validation of a Nomogram for Cognitive Impairment in Elderly Patients With Diabetes

**DOI:** 10.1155/2024/5583707

**Published:** 2024-08-19

**Authors:** Sensen Wu, Dikang Pan, Hui Wang, Julong Guo, Fan Zhang, Yachan Ning, Yongquan Gu, Lianrui Guo

**Affiliations:** Department of Vascular Surgery Xuanwu Hospital Capital Medical University, 45 Changchun Street, Xicheng District, Beijing, China

**Keywords:** cognitive impairment, diabetes, prediction model

## Abstract

**Objective:** The purpose of this study is to establish a predictive model of cognitive impairment in elderly people with diabetes.

**Methods:** We analyzed a total of 878 elderly patients with diabetes who were part of the National Health and Nutrition Examination Survey (NHANES) from 2011 to 2014. The data were randomly divided into training and validation cohorts at a ratio of 6:4. The least absolute shrinkage and selection operator (LASSO) logistic regression analysis to identify independent risk factors and construct a prediction nomogram for cognitive impairment. The performance of the nomogram was assessed using receiver operating characteristic (ROC) curve and calibration curve. Decision curve analysis (DCA) was performed to evaluate the clinical utility of the nomogram.

**Results:** LASSO logistic regression was used to screen eight variables, age, race, education, poverty income ratio (PIR), aspartate aminotransferase (AST), blood urea nitrogen (BUN), serum uric acid (SUA), and heart failure (HF). A nomogram model was built based on these predictors. The ROC analysis of our training set yielded an area under the curve (AUC) of 0.786, while the validation set showed an AUC of 0.777. The calibration curve demonstrated a good fit between the two groups. Furthermore, the DCA indicated that the model has a favorable net benefit when the risk threshold exceeds 0.2.

**Conclusion:** The newly developed nomogram has proved to be an important tool for accurately predicting cognitive impairment in elderly patients with diabetes, providing important information for targeted prevention and intervention measures.

## 1. Introduction

According to the ninth edition of the International Diabetes Federation (IDF) report, the global prevalence of diabetes was 9.3% (approximately 463 million people) in 2019. It is projected to increase to 10.2% (578 million people) by 2030 and further rise to 10.9% (700 million people) by 2045 [1]. Diabetes frequently results in various complications related to brain function, such as cognitive decline and depression. Several studies had shown that individuals with diabetes, both Type 1 and Type 2, were at a higher risk of experiencing cognitive decline and developing dementia compared to those without diabetes [2–5].

Impairments in cognitive function often leads to deficits in multiple cognitive domains, such as language, computation, judgment, memory, and executive function. These deficits can result in behavioral, emotional, and personality abnormalities, ultimately leading to a reduced ability to work and perform daily tasks. Cognitive impairment not only affects the overall health of individuals but also their capacity to live independently and be productive, placing substantial demands on social, hospital, and financial resources. This places a significant financial burden and psychological stress on both the family and society [6, 7]. Diabetes-related cognitive impairment is often overlooked during diagnosis and treatment, leading to long-term memory loss in many cognitively impaired diabetics [8]. Given the increasing prevalence of diabetes and its associated complications, it is crucial to establish a cognitive impairment prediction model specifically for elderly patients with diabetes. This recognition helps to improve the quality of life for patients with cognitive impairment and reduce the costs associated with long-term complications [9].

In oncology research, nomogram drawings have proven to be a useful tool in determining cancer prognosis [10]. However, there are fewer clinical prediction models available for assessing the risk of cognitive impairment in elderly patients with diabetes. In this study, we utilized the National Health and Nutrition Examination Survey (NHANES) database to establish and validate a nomogram to predict the risk of cognitive impairment in elderly diabetic individuals. This approach of early identification of patients at risk for cognitive impairment and implementation of targeted interventions has the potential to significantly improve patient prognosis and reduce healthcare costs. Additionally, it has the potential to enhance overall quality of life and alleviate family and socioeconomic burdens.

## 2. Materials and Methods

### 2.1. Data Collection and Study Population

The NHANES (https://wwwn.cdc.gov/nchs/) is a cross-sectional survey being conducted by the Centers for Disease Control and Prevention (CDC) to estimate the nutritional and health status of the US population. The survey uses a stratified, multistage probability design to recruit a representative sample of the US population. Data were collected through structured interviews with individuals at home, health screenings at mobile health screening centers, and laboratory sample analysis [11].

**Figure 1:**
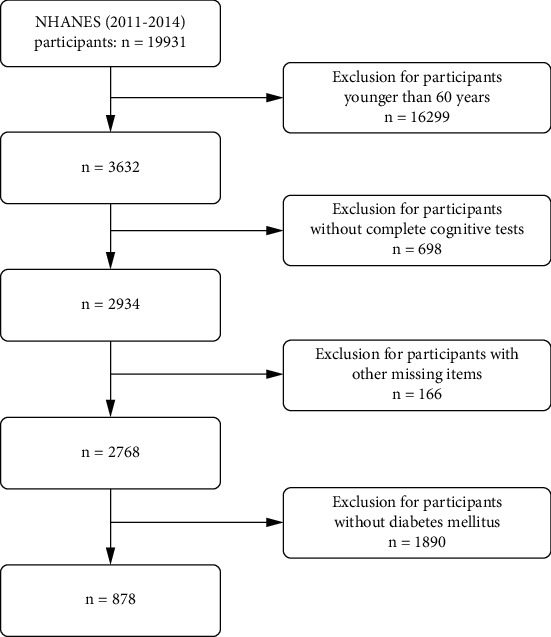
The screening process diagram in this study.

In this study, we analyzed data from NHANES from 2011 to 2014, which provides information on cognitive function. A total of 878 elderly patients with diabetes were included after screening, and the screening flow chart is shown in Figure 1. The diabetes was diagnosed according to the following standards: (1) fasting blood glucose (FBG) ≥ 7.0 mmol/L (126 mg/dL) or glycated hemoglobin (HbA1c) ≥ 6.5%, (2) diagnosis by a physician, and (3) use of antidiabetic medication.

### 2.2. Assessment of Cognitive Performance

In the 2011–2014 NHANES, a series of tests was administered to assess cognitive function in participants aged 60 years or older. Trained interviewers conducted assessments at the end of a face-to-face private interview at a mobile examination center. The cognitive function of participants was evaluated using three tests: the Consortium to Establish a Registry for Alzheimer's Disease Word Learning subtest (CERAD W-L) to assess immediate and delayed recall of new verbal information; the Animal Fluency Test (AFT) to measure categorical verbal fluency; and the Digit Symbol Substitution Test (DSST) to evaluate processing speed, sustained attention, and working memory. These tests have been widely utilized in mass screening efforts and clinical studies [12–14]. Although cognitive assessments cannot substitute for a clinical examination-based diagnosis, they are valuable in investigating cognitive function in relation to various diseases and risk factors.

The CERAD W-L assesses immediate and delayed learning ability for new verbal information (memory subdomain) [15]. The test consists of three consecutive learning trials and a delayed recall test. During the learning trials, participants were instructed to verbally read aloud 10 unrelated words, presented one at a time. Immediately after the presentation, participants were asked to recall as many words as possible. The order of the 10 words was changed in each of the three learning trials. The maximum achievable score on each trial was 10. Participants who were unable to read, either due to literacy issues or visual impairment, were instructed to repeat each word after it was read out by the interviewer. The delayed word recall test took place after the completion of AFT and DSST, approximately 8–10 min from the start of the word learning trials.

The AFT examines categorical verbal fluency, a component of executive function. The test demands awareness (e.g., naming animals), regardless of cultural context, that is not absolutely reliant on formal educational experiences of a particular culture [16]. The test has been demonstrated to distinguish between individuals with cognitive impairment and those with normal cognitive function [13, 14]. Participants are asked to name as many animals as possible in 1 min. A point is given for each named animal. In NHANES, participants first were asked to name three items of clothing as a practice test. Participants who could not name three articles of clothing did not continue with the AFT.

The DSST is a performance module of the Wechsler Adult Intelligence Scale (WAIS III). It is designed to assess processing speed, sustained attention, and working memory. The exercise is conducted using a paper form that has a key at the top containing nine numbers paired with symbols. Participants are given 2 min to copy the corresponding symbols into the 133 boxes that are adjacent to the numbers. The score is calculated based on the total number of correct matches. In NHANES, a sample practice test is administered prior to the main test, allowing participants to familiarize themselves with the task. Participants who were unable to correctly match the symbols with the numbers during the pretest practice were not continued.

There is no gold standard for the use of the CERAD, AFT, and DSST tests in identifying cognitive impairment, we conducted a combined analysis of previous studies [17, 18]. To minimize the influence of age on cognitive function, we divided the participants into three age groups: 60–69 years, 70–79 years, and 80 years or older. We used the lowest quartile of test scores in each group as a threshold to define cognitive impairment. In the three age groups, the lowest quartiles of CERAD scores were 21, 20, and 17; similarly, the lowest quartiles for the AFT were 13, 11, and 11, and the lowest quartiles for the DSST test were 33, 27, and 25, respectively.

### 2.3. Assessment of Covariates

Standardized questionnaires were collected on participants' sociodemographic characteristics, smoking status, diabetes drug use, hypertension, hypercholesterolemia, past history. Participants who smoke less than 100 cigarettes in their lifetime are classified as nonsmokers, while those who previously smoked more than 100 cigarettes but did not quit are defined as current smokers. Previous smokers were those who used to smoke more than 100 cigarettes but had already quit. Race/ethnicity is classified as Mexican American, other Hispanic, non-Hispanic White, non-Hispanic Black, and other races. Education level in our research is classified as lower than high school (less than 9th grade), high school education (include 9–12th grade [GED or equivalent]), or college or above (some college or associate's degree and college graduate or above). Marital status is classified into three categories in our researchers, the first being married or living with partners; the second being married, divorced, or separated; and the third being unmarried. Poverty income ratio (PIR) scores were defined as less 1, 1–3, and more than 3. Body mass index (BMI) is defined as weight (kilograms)/height^2^ (square meters) (normal: ≤ 25 kg/m^2^, overweight: 25–30 kg/m^2^, and obese: ≥ 30 kg/m^2^). It is calculated by dividing the household income by the poverty guidelines of a specific survey year [17, 19]. Similarly, laboratory indicators were included in our study. In order to increase the practicality of the predictive model, we have used as many variables as possible that can be obtained; in our study, the selection of variables was based on a combination of existing literature, clinical relevance, and preliminary analyses.

### 2.4. Statistical Analysis

All statistical analyses were performed using R software (Version 4.3.1). The data collected from the NHANES database were randomly divided into training and validation cohorts at a ratio of 6:4, and the variables were compared. Nonnormal data were presented as median (interquartile ranges). For categorical variables, the chi-square test or Fisher's exact test was used in the univariate analysis, while the *t*-test or rank-sum test was used for continuous variables. In the training cohort, least absolute shrinkage and selection operator (LASSO) regression was employed to select variables for the final model. The optimal regularization parameter (lambda) was determined through cross-validation, and variables with nonzero coefficients were retained. Clinical relevance and model parsimony were considered in the selection process, and the final model's performance was evaluated using cross-validation and external validation techniques. To validate our predictive model, we employed the area under the receiver operating characteristic (ROC) curve and calibration curves. The area under the curve (AUC), calculated using 10-fold cross-validation, assessed the model's discriminatory power, with an AUC of 1.0 indicating perfect discrimination. Calibration curves were used to compare predicted probabilities with observed outcomes, complemented by the Hosmer–Lemeshow test for goodness-of-fit. Additionally, we used bootstrapping (1000 samples) for internal validation, providing stable performance estimates and assessing potential optimism in the model's performance. Decision curve analysis (DCA) was conducted using the “rmda” package in R. The analysis involved calculating the net benefit for a range of threshold probabilities to evaluate the clinical utility of the predictive model. The net benefit was determined by considering true positives and false positives, with an appropriate weighting of false positives relative to false negatives. The results were plotted to compare the net benefit of the model against the treat-all and treat-none strategies, with higher net benefit curves indicating better clinical utility. Results with a *p* value of < 0.05 were considered statistically significant.

## 3. Results

### 3.1. Baseline Characteristics

The final study sample consisted of 878 participants.  Table 1 presents the baseline characteristics of the study participants in both the training cohort and the internal validation cohort. The sex distribution was similar between the two cohorts, with no significant difference observed (*p* = 0.465). Similarly, there were no significant differences in the distribution of age, race, education, PIR, BMI, and laboratory indicators such as albumin (ALB), blood urea nitrogen (BUN), total cholesterol (TC), serum creatinine (SCr), FBG, serum uric acid (SUA), triglyceride (TG), and others. There is still no significant difference between the two groups in the diseases such as coronary heart disease, hypertension, and hyperlipidemia (all *p* > 0.05). Overall, these findings suggest that the two cohorts were largely comparable in terms of baseline characteristics, except for marital status (*p* = 0.028) and HbA1c levels (*p* = 0.014).

### 3.2. Predictive Model

**Figure 2:**
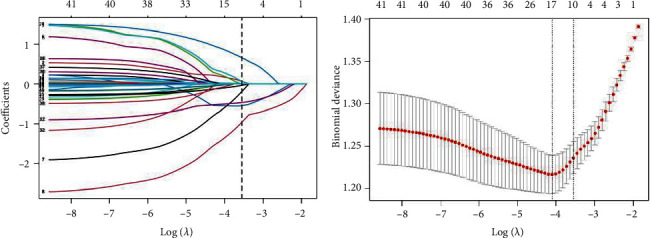
LASSO regression analysis in the training cohort. The coefficient profile is plotted in (a) and cross-validated error plot of the LASSO regression model is shown in (b).

The candidate predictors, sex, age, race, education, marital status, PIR, BMI, ALB, ALT, aspartate aminotransferase (AST), ALK, BUN, serum calcium, creatine kinase (CK), TC, SCr, FBG, Fe, Pi, TBIL, SUA, TG, smoking, drug use for DM, heart failure (HF), CAD, stroke, hypercholesterolemia, hypertension, HDL, HbA1c, depressed, somnipathy, and aspirin, were included in the original model, which were then reduced to eight potential predictors using LASSO regression analysis performed in the training cohort. LASSO coefficient profile (Figure 2(a)) shows the path of the coefficients for each predictor variable as the regularization parameter (lambda) changes. The *x*-axis represents the log-transformed values of lambda, while the *y*-axis shows the coefficients. Each line corresponds to a single variable, with coefficients shrinking to zero as lambda increases, indicating variable selection. Cross-validated error plot (Figure 2(b)) shows the mean squared error (MSE) from cross-validation for different values of lambda. The *x*-axis represents the log-transformed values of lambda, and the *y*-axis shows the cross-validated MSE. Error bars indicate the standard error of the MSE. The vertical line marks the lambda value that minimizes the MSE, representing the optimal balance between model complexity and predictive accuracy.

### 3.3. Nomogram Development and Validation

**Figure 3:**
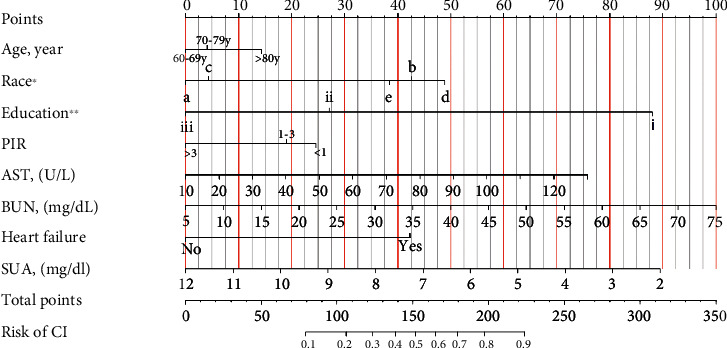
The nomogram of cognitive impairment in elderly patients with diabetes. Note: ^∗^a = Mexican American, b = other Hispanic, c = non-Hispanic White, d = non-Hispanic Black, e = other race—including multiracial. ^∗∗^i = lower than high school (less than 9th grade), ii = high school education (include 9–12th grade, GED or equivalent), iii = college or above (include some college or associate's degree and college graduate or above). PIR = poverty income ratio, AST = aspartate aminotransferase, BUN = blood urea nitrogen, SUA = serum uric acid.

**Figure 4:**
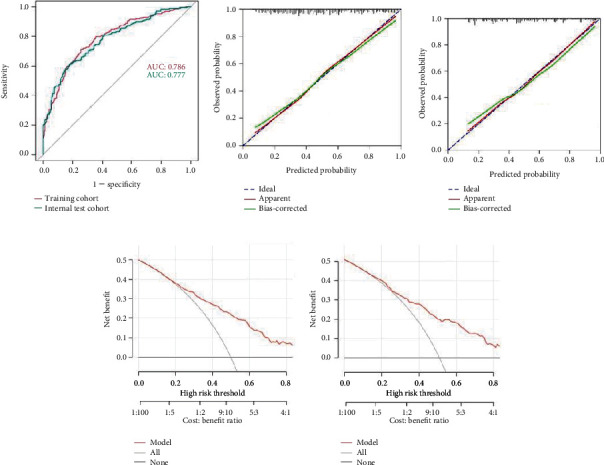
Validation of nomogram and DCA curves. (a) ROC curves for training and validation cohorts. (b, c) The calibration plots of the nomogram in the training and validation cohorts. The DCA curves of (d) the training cohort and (e) the validation cohort.

The final logistic model included eight independent predictors (age, race, education, PIR, AST, BUN, SUA, and HF) and was developed as a simple-to-use nomogram, which is illustrated in Figure 3. ROC curves for training set and validation set were used to evaluate the discriminability of the model. The results of ROC analysis showed that the AUC of the training set was 0.786 and the AUC of the validating set was 0.777, indicating that our model has good stability and prediction accuracy. The results are displayed in Figure 4(a).

The internal validation and calibration of the nomogram were performed using 1000 bootstrap analyses. The calibration plots of the nomogram in the different cohorts are plotted in Figures 4(b) and 4(c), which demonstrate a good correlation between the observed and predicted cognitive impairment. The results showed that the original nomogram was still valid for use in the validation sets, and the calibration curve of this model was relatively close to the ideal curve, which indicates that the predicted results were consistent with the actual findings.

### 3.4. Decision Curve Analysis

The net benefit curves for our predictive model, the treat-all strategy, and the treat-none strategy were plotted (Figures 4(d) and 4(e)). The *x*-axis represents the threshold probability, while the *y*-axis represents the net benefit. A higher net benefit curve indicates better clinical utility. If the net benefit of our model is higher than both the treat-all and treat-none strategies across a range of threshold probabilities, it suggests that our model provides a greater benefit in clinical decision-making. The points where the model's net benefit curve is above the treat-all and treat-none strategies indicate the range of threshold probabilities where the model would be most useful.

## 4. Discussion

This study conducted a comprehensive analysis of data from the NHANES 2-year cycle (2011–2012, 2013–2014) and found that lower education levels, lower PIR, and race were related risk factors. At the same time, cognitive function of patients with diabetes combined with cardiac insufficiency will also be affected. Additionally, some clinical biomarkers have been studied as potential predictive factors, including AST, BUN, and SUA levels. These markers may reflect potential metabolic disorders and inflammation, which are related to cognitive decline in elderly patients with diabetes.

Based on this, we developed and validated a predictive nomogram that can be easily used to predict cognitive decline in the elderly patients with diabetes. In our study, the ROC analysis of our training set showed an AUC of 0.786. After internal verification, the AUC of the validation set was found to be 0.777, and the calibration curve accurately depicts the calibration results of both the training group and the validation group, indicating that our model effectively predicts the risk of cognitive impairment in elderly individuals with diabetes. In our DCA, we found that within the threshold probability range of greater than 0.2, the DCA curve is located above the “none” and “all” reference lines, indicating that the performance of the model is acceptable within this range.

Although the mechanisms underlying changes in brain structure and function with age are still unclear, multiple studies [20–22] have shown that various brain functions are influenced by multiple factors that lead to cognitive decline. The decline in cognitive ability accelerates with age and can already be detected in middle age (45–55 years old) [23]. In our study, to reduce age-related factors, we divided the included population into three different age groups and calculated the lowest quartile for cognitive testing scores in each group. However, in our statistical analysis, we found that despite excluding some possible biases, the impact of age on cognitive function can still be observed. Therefore, it is necessary to routinely conduct cognitive function screening for the elderly population.

Multiple previous studies have indicated differences in cognitive function among various races and ethnicities [24–26]. One study [27] examined individuals over the age of 90 from different racial backgrounds to assess their cognitive abilities. The results revealed that Black people had the highest prevalence of cognitive impairment (57.4%), while Asian people had the lowest prevalence (32.7%). Another study [28] focused on examining the differences in cognition between Hispanic, non-Hispanic Black (NHB), and non-Hispanic White (NHW) older adults in the United States. Surprisingly, it found that Hispanic Americans had higher cognitive abilities than NHB individuals across all age groups. However, these studies did not observe any impact on the prevalence of cognitive impairment by race or ethnicity after adjusting for age, gender, and education. Additionally, recent studies have revealed that cognitive levels among different races can change significantly after immigration, suggesting that race may merely serve as a reflection of an individual's socioeconomic status [29]. Further analysis is necessary to better understand the specific impact in the future.

Cognitive inequality among the elderly is largely determined by disparities in early education [30, 31]. Moreover, cognitive scores tend to increase as education levels rise [28]. A multinational study [32] on global aging and adult health, utilizing consolidated data from the World Health Organization, the Health and Retirement Study, and the European Health, Aging, and Retirement Survey, examined over 85,000 individuals aged 50 and above. The study revealed that the impact of education on cognitive function is twice as significant as the impact of income. Additionally, education indirectly regulates the influence of income on cognitive function and may even counteract the negative effects of low-income lifestyles on cognitive health. Although cognitive testing methods may have varying effects on populations with different educational backgrounds, the measurement methods employed in the NHANES database have minimized the influence of education as much as possible. Nevertheless, the study still uncovered that education level remains a critical factor in determining cognitive function. Therefore, striving for universal education is essential in narrowing the cognitive gap caused by low income and early adverse conditions.

PIR—the ratio of family income to the poverty threshold—was used to define income. Lower PIR is linked to heightened levels of stress, limited access to healthcare, and diminished overall health. These factors may contribute to cognitive impairment in individuals with diabetes. A study [33] conducted in South Korea found that continuous access to social pension benefits can improve the cognitive abilities of elderly individuals. The provision of social pension can lead to improvements in nutrition, utilization of healthcare services, and engagement in physical activity, while also reducing economic pressure and associated stress levels. Findings from a social survey conducted in Malaysia revealed a positive correlation between financial status and both life satisfaction and cognitive function [34]. Similarly, a social survey [35] conducted in China suggested that urbanization may have a cumulative beneficial impact on cognitive function. Rural populations that have undergone planned urbanization in China demonstrated higher cognitive scores compared to nonmobile rural populations, with improvements in living conditions and changes in income being the main driving factors.

The interaction between the heart and the central nervous system holds clinical and pathophysiological significance, and it is estimated that 25%–80% of HF patients experience cognitive impairment [36]. A meta-analysis [37] on HF and cognitive impairment revealed an odds ratio of 1.67 (95% CI 1.15–2.42) for cognitive impairment in individuals with HF. The REGARDS study [38], a longitudinal cohort study, found that 14.9% of patients with HF also had cognitive impairment. These findings suggest that healthcare providers should consider evaluating cognition function when diagnosing HF.

A recent study [39] conducted on animals demonstrated that certain markers such as CK, lactate dehydrogenase, AST, and BUN showed elevated levels in animal models with cognitive impairment. This suggests that potential hematological markers can be further investigated as indicators of increased risk of cognitive impairment. In our study, we have identified three hematological markers, namely, AST, BUN, and SUA, that are associated with cognitive impairment.

The liver serves as the central metabolic hub of the human body, and the easiest way to monitor changes in liver function is through the examination of liver enzymes such as AST and ALT [40]. Many studies have also shown that alterations in liver enzymes can be indicative of cognitive function. For instance, a study [41] investigating the risk factors for cognitive impairment in patients after cardiac surgery identified elevated AST levels as an independent risk factor for postoperative cognitive impairment. Similarly, a survey conducted in China revealed a correlation between changes in AST levels and cognitive impairment [42]. Numerous studies have demonstrated a strong association between chronic kidney disease (CKD) and cognitive impairment, with various factors like vascular and metabolic issues contributing to the development of cognitive impairment in CKD [43]. BUN is a hematological marker observed in CKD patients, but its impact on cognitive function requires further investigation.

The relationship between SUA and cognitive function is contradictory [44]. It has been recognized as an independent cardiovascular risk factor in most studies. However, it is also a powerful antioxidant, and studies have shown that uric acid may actively participate in neurotoxicity and neuroprotective effects. These effects may be caused by oxidative stress or inflammatory processes in the central nervous system, or by other physical or systemic diseases [45]. In a large study with a substantial sample size, it was found that participants with higher SUA levels had a lower risk of cognitive impairment (hazard ratio: 0.78) [46]. However, it is important to note that uric acid can be a double-edged sword. High SUA levels in elderly patients increased the burden of cerebrovascular disease, thereby increasing the risk of vascular dementia [47]. Studies have indicated that the relationship between SUA levels and neurological diseases may follow a U-shaped pattern [48]. Further research is needed to elucidate the physiological and pathological role of SUA in cognitive impairment and its related thresholds.

The cognitive impairment associated with diabetes is a result of changes in the central nervous system [49]. The mechanism of cognitive impairment in diabetics is currently unclear, but it may be caused by endothelial cell dysfunction, oxidative stress, and chronic inflammation, which ultimately damage and kill neurons and synapses. These multiple mechanisms may act together to disrupt the normal signaling mechanisms involved in cognition, leading to cognitive decline in diabetics [50–52]. High glucose concentrations have been shown to cause leakage of the endothelial cell permeability barrier and increase the production of reactive oxygen species (ROS) in endothelial cells [50, 51]. In addition, studies have found that compared to normal conditions, the cognitive ability of zebrafish under high glucose conditions decreases. Oxidative stress plays a key role in this, and the use of vitamin D antioxidants can reduce cognitive decline under high blood sugar conditions to some extent [52].

The prevention of cognitive impairment is not a complete treatment for diabetics, but rather controlling blood sugar levels within the optimal range. Mone et al. found that compared to diabetics with normal blood sugar levels, those with high blood sugar had significantly impaired cognitive function as measured by MoCA scores. Additionally, a strong correlation was found between MoCA scores and blood glucose levels [53]. It cannot be overlooked that insulin resistance is also present in the diabetic population. The insulin signaling pathway mediates its effects on synaptic neurotransmission, neuronal and glial metabolism, and neuroinflammatory responses in the brain [54]. Insulin resistance increases the risk of atherosclerosis, endothelial dysfunction, and oxidative stress, and it can also affect mitochondrial function, thereby further affecting oxidative stress processes and increasing the risk of cognitive impairment in diabetics [55, 56]. Mone et al. have confirmed a correlation between insulin resistance and cognitive function [57]. Unfortunately, the indicators such as blood glucose status and insulin resistance were not obtained and validated in our research. We hope that there will be more comprehensive and forward-looking designs to focus on these indicators in the future and provide more comprehensive intervention guidelines for preventing cognitive function changes in diabetics.

This study is aimed at identifying the most critical predictive factors for cognitive impairment in individuals with diabetes. The findings will contribute to our understanding of the risk factors associated with this complication and may inform the development of targeted interventions to mitigate cognitive decline in this population. Furthermore, the use of nomogram drawing will provide a robust and practical method for predicting cognitive impairment in clinical practice. First, it provides clinicians with a quantitative tool that is more accurate in prediction compared to traditional methods and contributes to better risk stratification. Additionally, early identification of at-risk populations through this nomogram enables timely interventions that have the potential to improve patient quality of life and potentially reduce morbidity and mortality.

### 4.1. Limitations

Although our study used a large, representative sample of older Americans, and the NHANES database offers significant advantages in terms of survey methodology and quality control, this article still has some limitations. First, the cohort is based on a population from the US CDC, which may not be representative of the wider population, particularly those in low-income countries. Additionally, our model may include potential unmeasured confounders, such as diet and exercise. This omission limits the comprehensiveness of our analysis and may affect the overall results. Third, because the NHANES variable database is huge, it is impossible to include all the covariates related to diabetes, such as insulin resistance, blood glucose control, and follow-up, so the results may be affected by the loss of some important variables. Lastly, our study lacks external validation. Future studies should aim to externally validate our nomogram in different populations and settings.

## 5. Conclusion

The newly nomogram incorporates various prognostic variables and has been proven to be an important tool for accurately predicting cognitive impairment in elderly patients with diabetes, providing important information for targeted prevention and intervention.

## Figures and Tables

**Table 1:** Patient demographics and baseline characteristics.

**Characteristic**	**Training cohort** ^a^ **N** = 527	**Internal test cohort** ^a^ **N** = 351	**p** **value**
Man	288 (55%)	183 (52%)	0.465
Age (year)			0.983
60–69	281 (53%)	185 (53%)	
70–79	168 (32%)	113 (32%)	
≥ 80	78 (15%)	53 (15%)	
Race			0.296
Mexican American	54 (10%)	40 (11%)	
Other Hispanic	59 (11%)	37 (11%)	
Non-Hispanic White	204 (39%)	153 (44%)	
Non-Hispanic Black	149 (28%)	94 (27%)	
Others	61 (12%)	27 (7.7%)	
Educational status			0.870
Less than 9th grade	77 (15%)	52 (15%)	
High school education	217 (41%)	150 (43%)	
College or above	233 (44%)	149 (42%)	
Marital status			0.028
Married	307 (58%)	185 (53%)	
Widowed, divorced, or separated	184 (35%)	151 (43%)	
Unmarried	36 (6.8%)	15 (4.3%)	
PIR			0.905
< 1	106 (20%)	74 (21%)	
1–3	257 (49%)	172 (49%)	
> 3	164 (31%)	105 (30%)	
BMI (kg/m^2^)			0.760
≤ 25	94 (18%)	59 (17%)	
25–29.9	162 (31%)	116 (33%)	
≥ 30	271 (51%)	176 (50%)	
ALB (g/L)	42.0 (39.0, 44.0)	41.0 (39.0, 43.0)	0.390
ALT (U/L)	20 (16, 27)	19 (16, 25)	0.109
AST (U/L)	22 (19, 27)	22 (20, 27)	0.858
ALT/AST	1.10 (0.94, 1.33)	1.14 (0.97, 1.33)	0.072
ALP (U/L)	67 (55, 84)	67 (56, 83)	0.738
BUN (mg/dL)	16 (13, 21)	16 (12, 21)	0.669
SCa (mmol/L)	2.38 (2.30, 2.43)	2.35 (2.30, 2.43)	0.066
CK (U/L)	108 (72, 174)	102 (68, 161)	0.181
TC (mg/dL)	171 (144, 203)	167 (142, 207)	0.626
SCr (umol/L)	87 (72, 111)	86 (72, 107)	0.737
FBG (mmol/L)	7.27 (6.25, 9.02)	7.33 (6.00, 9.16)	0.533
Fe (umol/L)	13.1 (10.2, 16.5)	13.4 (10.4, 17.4)	0.255
Pi (mmol/L)	1.20 (1.07, 1.32)	1.20 (1.07, 1.32)	0.501
TBIL (umol/L)	10.3 (8.6, 12.8)	10.3 (8.6, 13.7)	0.092
SUA (mg/dL)	5.70 (4.90, 6.80)	5.90 (5.00, 7.00)	0.377
TG (mg/dL)	143 (102, 217)	148 (102, 222)	0.899
HbA1c (%)	6.70 (6.25, 7.50)	6.60 (6.00, 7.35)	0.014
HDL (mg/dL)	47 (40, 56)	46 (39, 56)	0.301
Smoking			0.917
Never	235 (45%)	157 (45%)	
Previous	226 (43%)	147 (42%)	
Current	66 (13%)	47 (13%)	
Drug for DM	431 (82%)	280 (80%)	0.457
HF	54 (10%)	46 (13%)	0.192
CAD	119 (23%)	81 (23%)	0.864
Stroke	54 (10%)	25 (7.1%)	0.113
Hypertension	387 (73%)	253 (72%)	0.658
Hypercholesterolemia	347 (66%)	225 (64%)	0.596
CKD	55 (10.4%)	39 (11.11%)	0.10
Depression	69 (13%)	44 (13%)	0.809
Sleep disorder	79 (15%)	67 (19%)	0.110
Aspirin	345 (65%)	217 (62%)	0.271

Abbreviations: ALB = albumin; ALP = alkaline phosphatase; ALT = alanine aminotransferase; AST = aspartate aminotransferase; BMI = body mass index; BUN = blood urea nitrogen; CAD = coronary artery disease; CK = creatine kinase; CKD = chronic kidney disease; FBG = fasting blood glucose; HbA1c = glycated hemoglobin; HDL = high-density lipoprotein; HF = heart failure; Pi = serum phosphorus; PIR = poverty income ratio; SCa = serum calcium; SCr = serum creatinine; SUA = serum uric acid; TBIL = total bilirubin; TC = total cholesterol; TG = triglyceride.

^a^Nonnormal data were presented as median (interquartile ranges); *n* (%).

## Data Availability

All data were included in NHANES database (https://www.cdc.gov/nchs/nhanes/index.htm). And the datasets used or analyzed during the current study are available from the corresponding authors on reasonable request.

## Nomenclature

AFT: Animal Fluency Test
ALB: albumin
ALP: alkaline phosphatase
ALT: alanine aminotransferase
AST: aspartate aminotransferase
AUC: area under the curve
BMI: body mass index
BUN: blood urea nitrogen
CAD: coronary artery disease
CK: creatine kinase
CKD: chronic kidney disease
DCA: decision curve analysis
FBG: fasting blood glucose
HbA1c: glycated hemoglobin
HDL: high-density lipoprotein
HF: heart failure
LASSO: least absolute shrinkage and selection operator
NHANES: National Health and Nutrition Examination Survey
Pi: serum phosphorus
PIR: poverty income ratio
ROC: receiver operating characteristic
SCa: serum calcium
SCr: serum creatinine
SUA: serum uric acid
TBIL: total bilirubin
TC: total cholesterol
TG: triglyceride
